# Clinical Utility of Biosensing Platforms for Confirmation of SARS-CoV-2 Infection

**DOI:** 10.3390/bios11060167

**Published:** 2021-05-24

**Authors:** Le Minh Tu Phan, My-Van Tieu, Thi-Thu Pham, Sungbo Cho

**Affiliations:** 1 Department of Electronic Engineering, Gachon University, Seongnam-si 13120, Korea; plmtu@smp.udn.vn; 2 School of Medicine and Pharmacy, The University of Danang, Danang 550000, Vietnam; ptthu@smp.udn.vn; 3 TST Trading Service Technology Co., Ltd., Hochiminh City 723000, Vietnam; myvantieucau@gmail.com; 4 Department of Health Sciences and Technology, GAIHST, Gachon University, Incheon 21999, Korea

**Keywords:** SARS-CoV-2, COVID-19 clinical diagnostics, nucleic acid amplification, RT-PCR, RT-LAMP, optical biosensor, lateral flow assay, ELISA, electrochemical biosensor, lab-in-a-tube

## Abstract

Despite collaborative efforts from all countries, coronavirus disease 2019 (COVID-19) pandemic has been continuing to spread globally, forcing the world into social distancing period, making a special challenge for public healthcare system. Before vaccine widely available, the best approach to manage severe acute respiratory syndrome coronavirus 2 (SARS-CoV-2) infection is to achieve highest diagnostic accuracy by improving biosensor efficacy. For SARS-CoV-2 diagnostics, intensive attempts have been made by many scientists to ameliorate the drawback of current biosensors of SARS-CoV-2 in clinical diagnosis to offer benefits related to platform proposal, systematic analytical methods, system combination, and miniaturization. This review assesses ongoing research efforts aimed at developing integrated diagnostic tools to detect RNA viruses and their biomarkers for clinical diagnostics of SARS-CoV-2 infection and further highlights promising technology for SARS-CoV-2 specific diagnosis. The comparisons of SARS-CoV-2 biomarkers as well as their applicable biosensors in the field of clinical diagnosis were summarized to give scientists an advantage to develop superior diagnostic platforms. Furthermore, this review describes the prospects for this rapidly growing field of diagnostic research, raising further interest in analytical technology and strategic plan for future pandemics.

## 1. Introduction

The coronavirus disease 2019 (COVID-19) pandemic has highlighted the importance of the prompt and sensitive diagnosis of viral infections to enable effective tracing and implementation of public health measures for the prevention and management of outbreaks. Severe acute respiratory syndrome coronavirus-2 (SARS-CoV-2) can transmit directly to human by droplets, causing the slight-to-severe symptoms of respiratory infections in humans [1,2]. In 2002 and 2012, there were two human coronaviruses transmitted from animals to human to cause acute respiratory disease with high pathogenicity including Severe Acute Respiratory Syndrome (SARS) and Middle East Respiratory Syndrome (MERS), and current highly pathogenic SARS-CoV-2 has been making the outbreak of the global COVID-19 pandemic since December 2019 [3,4,5]. In January 2020, China was the first country to confirm viral deaths, and the number of deaths exceeded 100, with infections increasing rapidly [6]. Countries worldwide are currently dealing with this virus, and at least 47 other countries and territories were declared infected with SARS-CoV-2 in February 2020 [7]. As the number of viral deaths worldwide surpassed 10,000, concern shifted to Europe, especially Italy [8]. At the end of March 2020, the number of confirmed cases of COVID-19 in Korea was approximately 10,000 due to a large outbreak in the city of Daegu 150 miles southeast of Seoul [9]. Simultaneously, in India, people were instructed to remain indoors unless there was an emergency [10]. A new milestone of 1,000,000 deaths was reached in September 2020. According to Johns Hopkins University, as of the 8th November this included 236,073 cases in the USA, 161,106 in Brazil, and 125,562 in India [11]. At present (November 7th 2020), the World Health Organization (WHO) states that there are currently 49,765,123 cases with 1,250,160 deaths and 35,315,721 patients recovered to date [12].

In late 2019, severe respiratory distress with pneumonia-like symptoms was reported in Wuhan, China. This virus, as of animal origin, was named SARS-CoV-2 or more commonly, the novel coronavirus, resulting in the acute respiratory infection as COVID-19 [13]. In the early stages, these undocumented viral infections often go undetected due to mild or limited symptoms. Their comparatively high ability to spread and high prevalence caused a large proportion of the global population to be exposed to the virus [14]. At present, there is no specific treatment but multiple effective vaccines being administered worldwide. Immediate action from both academia and industry has led to the development of multiple COVID-19 diagnostic systems that have gained rapid regulatory approval and have been used clinically since their establishment [15,16]. This has led to the development of powerful and accurate analytical tools that use biosensors for biomarker detection. Integrated approaches offer a better standpoint for the development of biosensors during the COVID-19 pandemic.

Based on international experience, most countries worldwide have identified the five following prerequisites for relaxing COVID-19 containment and restriction measures: knowledge of contagiousness of the infection, community participation, adequate health system capacity, and border control [17]. In this review, we present the importance of using biosensor systems and their effectiveness in COVID-19 diagnosis. Different types of biosensors and integrated techniques of biosensors with diverse potential applications are needed. Therefore, this review provides an overview of the different types of biosensors being used to detect SARS-CoV-2: optical sensing, electrical sensing, lab-in-a-tube sensing system, lab-on-a-chip sensing system, electrochemical sensing, and nucleic acid amplification (NAA) sensing technique. These biosensors have made huge contributions to the diagnosis and treatment of COVID-19.

## 2. Virology of SARS-CoV-2 and Its Specific Biomarkers for Diagnostics

**Figure 1:**
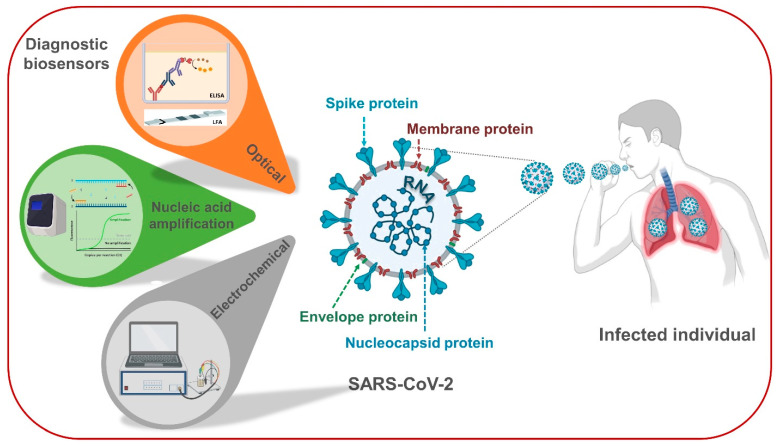
Structural models of SARS-CoV-2 and its developed diagnostic platforms. The subgenomic RNAs of the genome encode the following four main structural proteins: Spike protein (S), Envelope small membrane protein (E), nucleocapsid protein (N), and membrane protein (M), as well as several accessory proteins. Schematic representation of an ideal sensing platform composed of nucleic acid amplification technique, optical, and electrochemical sensing platforms. The general configuration of the different sensing platforms for SARS-CoV-2 detection is illustrated.

SARS-CoV-2 belongs to the genus *Betacoronavirus* (subgenus Sarbecovirus) of the family Coronaviridae and is an encapsulated positive-single-stranded RNA virus. Its capsid is encapsulated in a lipid bilayer, and the viral genome, not complementary sequences, encodes viral proteins [18,19]. To date, four coronaviruses (a, b, g, and d) have been identified: human coronavirus (HCoV)-229E, HCoV-NL63, HCoV-HKU1, and HCoV-OC43 [20,21]. The isolated novel b-CoV shows 88% homogeneity with sequences of two bat-derived coronaviruses—bat-SL-CoVZC45 and bat-SL-CoVZXC21—and shows approximately 50% sequence identity with the sequences of MERSPeer CoV. The SARS-CoV-2 genome has a length of 26–32 kb, which is the largest of all RNA viruses [18,21]. Infectious SARS-CoV-2 (diameter ∼100 nm) contains the 29,903 nucleotide RNA genome along with four structural proteins: spike (S), envelope small membrane (E), nucleocapsid (N), and membrane protein (M). The N proteins bind to the RNA genome in a helical, symmetrical manner, like particles on a string, and this genome structure is surrounded by a lipid bilayer that binds to the E, M, and S proteins [22,23,24]. The encapsulation of genomic RNA with structural proteins leads to the formation of new SARS-CoV. The diagnostic methods of COVID-19 confirmation are mainly based on NAA techniques for RNA detection of SARS-CoV-2 as well as optical and electrochemical diagnostic biosensors for SARS-CoV-2 protein antigens (Figure 1). Antigen expression then stimulates cellular and humoral immunity of the body mediated by virus-specific B and T cells. Similar to conventional acute viral infections, the antibody profile against SARS-CoV-2 follows a typical IgM and IgG production pattern. SARS-specific IgM antibodies disappear after 12 weeks, while IgG antibodies can persist for a long time, suggesting that SARS-specific IgG antibodies, mainly S- and N-specific antibodies, play a protective role in primary immune response against SARS [25]. Long et al.2020 studied the antibody response to SARS-CoV-2 infection in 285 patients with COVID-19 in China. Seroconversion for IgM and IgG occurred concurrently or sequentially, and the mean day of seroconversion for immunoglobulins was 13 days after the onset of COVID-19 symptoms. After 19 days of symptom attack, a 100% IgG seroconversion rate was noted [26]. Notably, antibody response and viral clearance may be delayed in immunocompromised individuals and those subsequently infected with SARS-CoV-2 [27]. Table 1 was created after reviewing the viral structure and immune response against SARS-CoV-2 to provide an overview of the advantages and disadvantages of its biomarkers as target analytes for applicable biosensors to confirm SARS-CoV-2 infection.

## 3. Case Studies of Biosensing Technologies for SARS-CoV-2 in Clinical

COVID-19 viral disease is officially global pandemic, currently accounting for the highest number of deaths worldwide. Special screening is extremely important as an effective way to monitor and manage the pandemic before reaching herb immunity through effective vaccination against SARS-CoV-2. A rapid population control task for COVID-19 has been documented using innovative methods in biosensor development [34]. Biosensors are selected as promising detection devices with enormous potential as point-of-care (POC) tools to confirm the SARS-CoV-2 infection. Timely testing also helps to effectively allocate medical resources and save time for frontline medical staff. Hence, simple, rapid, cost-effective, and accessible detection techniques as POC diagnostics for large-scale screening and field testing of SARS-CoV-2 infection is important and should urgently be expedited to control the rapid and contagious spread of COVID-19. The developed sensing platforms were summarized in Table 2 in term of target analytes, sensing performance, analytical/clinical sensitivity and specificity, and commercial products, including optical biosensor (enzyme-linked immunosorbent assay (ELISA) or lateral flow assay (LFA)), lab-in-a-tube sensing system, lab-on-a-chip system, electrochemical sensing, and NAA-based techniques. This table will give an overview of significant achievement in the current sensing platforms for the accurate diagnosis of COVID-19, suggesting the suitable selection of applicable approaches all over the world.

### 3.1. Nucleic Acid Amplification-Based Techniques as Gold Standard Diagnostic Tests

**Figure 2:**
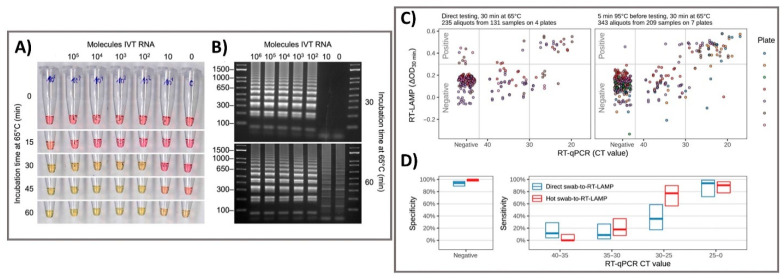
Colorimetric RT-LAMP and LAMP sequencing for the clinical detection of SARS-CoV-2 RNA. (**A**) The oligonucleotide set for the nucleocapsid (N) gene of SARS-CoV-2 was added to the RT-LAMP reaction and incubated at 65 °C. The colors of samples changed from red-to-yellow and the negative control was yellowish. (**B**) Gel electrophoresis showed RT-LAMP reaction products with distinct banding patterns. (**C**) For clinical pharyngeal swab samples, the direct swab-to-RT-LAMP assay measurements or after 5 min of heat treatment at 95 °C were compared for their ΔOD values from the swab-to-RT-LAMP assay and CT values from the RT-qPCR assay. (**D**) The sensitivity and specificity of the swab-to-RT-LAMP assay were revealed with their 95% confidence intervals, with the direct swab-to-RT-LAMP assay (blue color) and the heated swab-to-RT-LAMP assay (red color). Reprinted with permission from [41]. *Sci. Transl. Med.* 2020, 12, 556, eabc7075. Copyright 2020, American Association for the Advancement of Science.

A variety of NAA techniques have been incorporated into well-known clinical diagnostic tests, such as polymerase chain reaction (PCR), real-time PCR, and reverse transcription-mediated isothermal amplification (RT-LAMP). These techniques have widespread research applications in the diagnosis of COVID-19 as the gold standard diagnosis at the start of a pandemic. These techniques are well-developed owing to their simplicity, sensitivity, and speed. Chu et al. 2020 developed a one-step quantitative real-time RT-PCR in addition to a biological sensor for monitoring two different regions (ORF1b and ORFN) of the SARS-CoV-2 viral genome. Instead of two-step RT-PCR that separately conducts RNA reverse transcription and amplification steps, reverse transcriptase and DNA polymerase enzymes were premixed in a single tube that allows both steps to be performed in single reaction. These NAA-based sensors excel in the analytical requirements but are typically limited due to their time-consuming processes. RT-PCR platform has a lengthy laboratory workflow requiring multiple solution operation steps and relies on sophisticated equipment for thermal cycling and optical signal detection. It may not be a viable option for the screening of COVID-19 at locations where laboratories and highly trained technicians are absent. The aim of one-step quantitative RT-PCR is the rapid detection of SARS-CoV-2 in human samples (75 min), which is highly acceptable in clinical tests, however, it is more cost-effective, and required a robust manner in laboratories in different geographical regions. This two-stage amplification closed-tube diagnostic assay was used to test SARS-CoV-2 samples with significantly enhanced sensitivity compared to conventional RT-PCR with dynamic range of at least seven orders of magnitude (2 × 10^−4^–2000 TCID_50_/reaction) for RNA from SARS infected cells and below 10 copies per reaction for DNA plasmid as positive standards [38]. Chow et al. 2020 demonstrated the potential of the LAMP-based sensor of SARS-CoV-2 by monitoring the color change of the different concentrations of clinical samples which was detectable by naked eye [40]. This method was designed to directly amplify the target through one-step RT-LAMP test, then target RNA was confirmed via colorimetric method that showed the efficiency of detection of target RNA genome within range of 45–105 min including sample extraction time, depending on viral load. The tested clinical samples, which were different in nature (respiratory samples, nasopharyngeal swabs, sputum/deep throat saliva, and throat swabs), were confirmed by RT-PCR and collected at different reaction times (60 min and 90 min). Interestingly, the test with nasopharyngeal swab samples showed the highest sensitivity, with 96.88% (95% CI: 0.93–1.00) and 98.96% (95% CI: 0.97–1.00) of 96 samples positive by RT-LAMP at 60 and 90 min, respectively. In addition, the other clinical samples had high sensitivity of at least 93.33% (0.87–1.00), suggesting that this platform exhibits high potential in the detection application of SARS-CoV-2. In addition, Viet Loan et al. 2020 investigated key issues related to the colorimetric RT-LAMP assay and LAMP-sequencing, sensor characteristics, sensitivity and specificity in detecting SARS-CoV-2 RNA both in vitro and in vivo. The inclusion of RT-LAMP and colorimetric methods in the matrix increased the simple, scalable, and broadly applicable testing methods of the sensor. Furthermore, the sensor characteristics of the colorimetric RT-LAMP assay were tested on 768 pharyngeal swab specimens using a primer set specific for the N gene and compared an RT-PCR assay using a sensitive primer set with a sensitivity of 97.5% and specificity of 99.7%. In particular, the swab-to-RT-LAMP assay without a prior RNA isolation step showed excellent specificity (99.5%) but lower sensitivity (86% for CT < 30) than the RT-LAMP assay [41] (Figure 2). Furthermore, Ackerman et al. 2020 developed combinatorial arrayed reactions for multiplexed evaluation of nucleic acids (CARMEN) for the evaluation of multiplex pathogenic nucleic acids to detect pathogens. CRISPR-based nucleic acid detection reagents containing in nanoliter droplets could self-organize in arrays to pair with droplets of amplifies samples, testing samples against CRISPR RNA (crRNA) in replicate. With the combination of CARMEN and Cas13 detection, the assay simultaneously differentiated 169 human-related viruses and incorporated an additional crRNA to detect target of COVID-19. The CARMEN assay enables the scalability, miniaturization, and cost-effectiveness, shifting diagnostic ability from targeted high-priority samples to comprehensive large samples [69]. Traditional NAA technique is time-consuming and may have false-positive outputs based on the working experience of the technician with careful consideration throughout the testing time. However, PCR has become an indispensable and integral part of clinical and diagnostic research as the gold standard in hospitals due to their unique performance. One of the significant applications of the NAA-based techniques was their utility in the confirmation of SARS-CoV-2 infection for the better intervention of COVID-19 pandemic, which can shift diagnostic and surveillance efforts from targeted testing of high-priority samples to comprehensive testing of large sample sets, bringing great benefits to patients and public health.

### 3.2. Optical Sensing Platforms as Rapid Point-Of-Care Screening Tests

**Figure 3:**
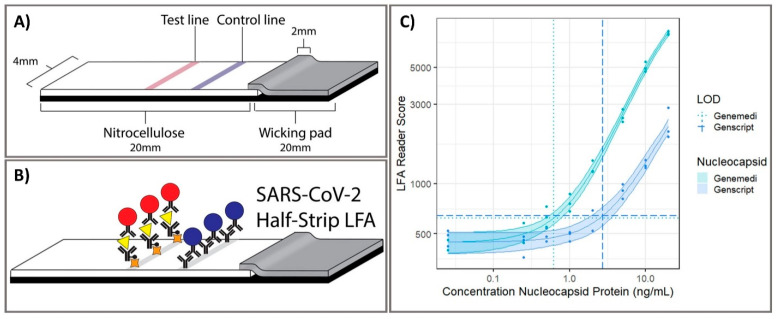
Point-of-care half-strip lateral flow assay for the detection of the nucleocapsid antigen of SARS-CoV-2. (**A**) The 4 mm width half-strip was constructed using a 20 mm nitrocellulose analytical membrane, 20 mm wicking pad by using a Kinematic Matrix guillotine cutter. (**B**) LFA was treated in buffer and color intensity of test zone was differentiated after 20 min. (**C**) The dosage response curve for half-band LFA using nucleocapsid proteins from two commercially available sources, measured with commercially available optical LFA readers. Reprinted with permission from [45]. *Anal. Chem*. 2020, 92, 16, 11305–11309. Copyright 2020, American Chemical Society.

Optical biosensors are one of the most common platforms that have been exploited to monitor various target for clinical diagnostics. They detect biological interactions by evaluating induced variations in the properties of light, such as intensity, wavelength, index of refraction, or polarization. Cutting-edge optical sensing platform technologies are currently being investigated for COVID-19 clinical samples, including samples based on lateral flow assays (LFAs), enzyme-linked immunosorbent assay (ELISA), chemiluminescent immunoassay, plasmonic biosensor, and localized surface plasmon resonance. Grant et al. 2020 developed half-strip LFAs as useful first step in the development of LFA platforms for the detection of SARS-CoV-2 using commercially available antibodies. This half-strip LFA exhibited high sensitivity toward SARS-CoV-2 with limit of detection (LOD) at 0.65 ng/mL by visual read or optical reader [45] (Figure 3). LFA is a good biosensing candidate for diagnostic applications owing to their advantages including high sensitivity and specificity, excellent biological compatibility, short duration, stable output, and affordability. More importantly, the LFA holds the potential for large-scale production and commercialization with convenient protocol without technical professions, allowing it to be POC test worldwide for initial screening of SARS-CoV-2 infection. Due to the feasibility of LFA biosensing platforms for the effective detection of SARS-CoV-2 biomarkers, there were several LFA strips developed from different companies. Demey et al. 2020 evaluated the sensing performance to detect SARS-CoV-2 using four immunochromatographic antibody assays of different commercial companies. Using SARS-CoV-2 positive samples confirmed by RT-PCR from 22 patients, they demonstrated that the ability of COVID-19 confirmation through antibody using these LFAs was depended on time with the median detection time about 8–10 days since the onset of symptoms, and the sensitivity was increased up to 60–80% on day 10 and 100% on day 15, indicating that these LFA tests were reliable at 14–15 days post-infection [50]. The low-cost LFA combined with an easily accessible synthetic biosensor that can function with bodily fluids as samples provides a comprehensive solution for the diagnosis of non-communicable diseases in resource-limited developing countries [70]. Instead of LFAs, the use of dual-functional plasmonic biosensor by combining plasmonic photothermal and localized surface plasmon resonance sensing transduction could provide a promising alternative optical biosensor. Qiu et al. 2020 identified an optical LOD of approximately 0.22 pM and confirmed a detection range of 0.01 pM to 50 μM for the detection of target SARS-CoV-2 sequences [56]. The highly sensitivive detection of target sequences was achieved by using 2D gold nanoislands (AuNIs) functionalized with complementary DNA receptors through nucleic acid hybridization. To enhance sensing performance, thermoplasmonic heat was generated on AuNI chip under illuminated at plasmonic resonance frequencies, elevating the in situ hybridization temperature and facilitating the accurate differentiation of two similar gene sequences. This dual-functional plasmonic biosensor exhibited the potential application in nucleic acid tests for viral disease diagnosis. Overall, these types of optical sensing platform enable the detection of SARS-CoV-2 biomarkers for the confirmation of COVID-19 in human samples including serum, plasma, blood, nasopharyngeal, and oropharyngeal swab specimens, with high sensitivity, specificity. With these advantages, the strong capability of translation of the signal intensity into the accurate concentration of biomarkers makes optical platforms important as applicable biosensors for POC diagnostic of COVID-19 in clinical. Therefore, almost clinical tests utilized optical sensing platform have been currently exploited as an effective tool for diagnosis of disease by sensing of biomarkers in human biological specimens. Compared to other optical biosensors that have been developed to detect SARS-CoV-2, it is worth noticing that LFA exhibits the superior potential to serve as diagnostic tools for initial screening of COVID-19 with acceptable results, short-time consumption and reasonable price, suggesting a good choice for developing and underdeveloped countries to utilize LFAs as POC diagnostic tool for managing the COVID-19 pandemic.

### 3.3. Lab-In-A-Tube and Electrochemical Sensors as Emerging Ultrasensitive Real-Time Monitors

**Figure 4:**
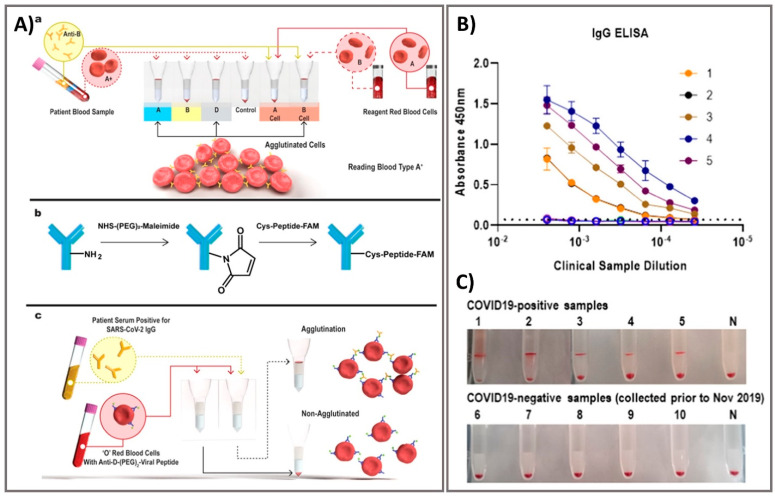
Rapid agglutination assays as serological testing for the detection of antibodies against SARS-CoV-2. (**A**) Schematic illustration of blood typing column agglutination test (CAT) with the brief antibody-peptide bioconjugates to produce the SARS-CoV-2 serological assay. (**a**) Pipette a mixture of reagent red blood cells (RRBCs) with patient samples onto a gel card containing separation media, followed by incubation of the card for 5–15 min. (**b**) The bioconjugation procedure to produce the antibody-peptide in two steps. (**c**) Antibody-peptide-coated RRBCs were incubated with a patient sample on a neutral gel prior to centrifugation to separate agglutinated RRBCs from free RRBCs for visual examination. Following optimization of the gel card assays to distinguish between SARS-CoV-2-positive samples and negative controls, 10 clinical samples were tested in both gel cards and by indirect IgG ELISA. (**B**) The results of indirect IgG ELISA comparing 10 samples, including PCR-confirmed SARS-CoV-2-positive samples and samples from healthy individuals collected before the SARS-CoV-2 outbreak. (**C**) Digital images of gel card assays recorded from experiments could identify positive/negative of antibodies, negative control noted (“N”). Reprinted with permission from [62]. *ACS Sens.* 2020, 5, 8, 2596–2603. Copyright 2020, American Chemical Society.

**Figure 5:**
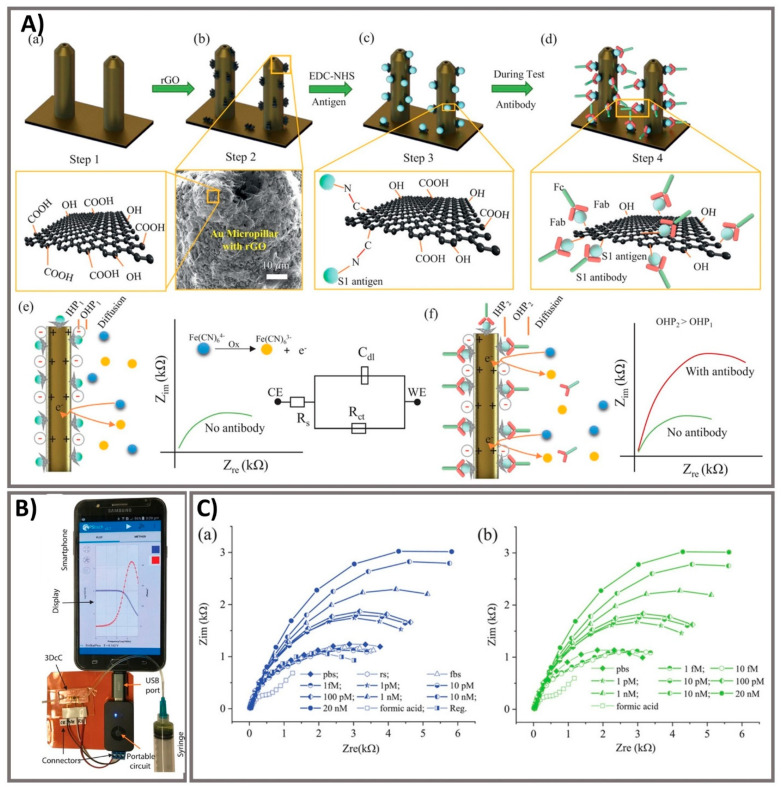
Ultra-rapid electrochemical immunosensor using aerosol jet nanoprinted reduced graphene oxide-coated 3D electrode for the detection of antibodies against SARS-CoV-2. (**A**) Functionalization of the 3D electrode and sensing operation. (**a**) AJ-printed gold micropillars prior to the surface treatment. (**b**) Coating rGO sheets onto the electrodes. (**c**) Immobilization of viral antigens onto rGO sheets. (**d**) Selective binding of antibody with specific antigens after introduction. (**e**) Schematics showing the sensing principle of the 3DcC device. (**f**) Schematic illustration of the Nyquist plot alternation via electrical impedance spectroscopy (EIS) before and after antibody introduction and binding with the antigens on the electrode surface. (**B**) The connection of the 3DcC device interfaced with a portable potentiostat to a smartphone via a USB-C connection for signal recording. (**C**) Sensing performance of antibodies against SARS-CoV-2 spike S1 antigen at different molar concentrations from 1 fM to 20 nM in (**a**) PBS solution and (**b**) after sensor regeneration using low-pH chemistry. Reprinted with permission from [65].

Laboratory diagnostics which compatible with critical laboratory equipment are conceptually easily applied to patients, given the features of the following multiple operations of virus testing [71]. Exposure tracking can limit the viral spread, however, population screening to determine virus infection levels in the community is a longer-term need. For lab-in-a-tube, Alves et al. 2020 built a sensing platform using gel tag agglutination tests to target SARS-CoV-2 with rapid case identification (Figure 4). Ten serological samples in both gel cards and indirect IgG ELISA were tested and showed that similar performance between them, suggesting this assay as one of suitable approach for clinical diagnosis compared to conventional ELISA, owing to its advantages in excellent resolution and benefits of high throughput, high speed (10–30 min), automatic in most cases, and possibility for POC diagnostics. From gel card agglutination assays to lab-in-a-tube systems, this simple, rapid, and scalable approach could identify disease-specific activity to apply in SARS-CoV-2 testing, suggesting the ability to move diagnostics to the POC test for COVID-19 confirmation [62]. In addition, Lin et al. 2020 recently demonstrated real-time and continuous monitoring platform using integrated diagnostic microchips, homemade mobile fluorescence detectors, and microfluidic immunoassay systems for the simultaneous detection of IgG/IgM/antigen in SARS-CoV-2. This system was utilized for SARS-CoV-2 serological testing, displaying high accuracy not only in distinguishing between infected and uninfected cases but also in determining the severity the disease, allowing disease staging as follows: stage 1 (infected 1–7 days), stage 2 (infected 8–14 days), and stage 3 (infected over 14 days). Furthermore, this system showed excellent sensor characteristics with a rapid response time of 15 min [63]. For case of electrochemical sensor, Vadlamani et al. 2020 designed an electrode composed of cobalt-functional TiO_2_ nanotubes (Co-TNT) as electrochemical sensor for the rapid detection of SARS-CoV-2 at low concentration range from 14–1400 nM with LOD of ~0.7 nM. The authors then used this system for real-time concentration measurements, showing a linear response in detecting viral proteins within concentration range for approximately 30 s in saliva and nasal secretions. The sensitivity of this approach can also be improved by using longer Co-TNTs due to higher surface area results in higher response rates, thus higher electric current can be obtained even at lower protein concentrations [64]. Furthermore, an advanced nanomaterial-based electrochemical biosensor to detect SARS-CoV-2 antibodies within seconds was successfully developed to enhance the rapid diagnosis of COVID-19 for the better treatment and prevention of diseases [65]. The three-dimensional (3D) electrodes were printed using 3D nanoprinting and were coated with nanoflakes of reduced-graphene-oxide (rGO); specific viral antigens were then immobilized on the rGO nanoflakes. The electrode was then integrated with a microfluidic device and applied as an electrical immunosensor. In the presence of antibodies against the SARS-CoV-2 S1 protein, the antibodies selectively bound to the antigens due to their strong immunoaffinity, leading to a change in impedance of the electrical circuit, which is detected via impedance spectroscopy. Antibodies to SARS-CoV-2 S1 protein and its receptor-binding domain were detected by a smartphone-based user interface within 10 s with a wide concentration range from 1 fM to 20 nM at LOD of 2.8 × 10^−15^ and 16.9 × 10^−15^ M, respectively (Figure 5). Seo et al. 2020 developed the field-effect transistor based biosensing device with the support of antibody functionalized graphene sheets to detect SARS-CoV-2 protein in human nasopharyngeal swab specimens. This platform helped improve nano-sensor performance in clinical samples for over 1 min without significantly altering the sensing capacity to detect SARS-CoV-2 without requiring sample pretreatment or labeling. This device showed good sensitivity for the detection of SARS-CoV-2 spike protein at the concentration of 1 fg/mL in phosphate-buffered saline and 100 fg/mL in clinical transport medium. Moreover, the device could detect SARS-CoV-2 in the culture medium with LOD of 1.6 × 10 pfu/mL and clinical samples with LOD of 2.42 × 10^2^ copies/mL, respectively. This fully reversible modular sensing platform is a viable candidate for continuous clinical monitoring [61]. Overall, these biosensing platforms mentioned above exhibits high sensitivity, selectivity, and the rapid ability of monitoring SARS-CoV-2 for application in diagnosis of COVID-19. However, it highly requires the technical professions and instruments to conduct the sensing process that could ensure the accurate diagnosis. The important mission for researchers is to achieve proper stability, remove unwanted noise, and make the products commercially applicable after conducting a successful clinical trial.

## 4. Concluding Remarks and Perspectives

The recent global COVID-19 outbreak has killed more than 1,669,982 people (as of 19 December 2020) and has strongly affected the global economy, causing economic hardship for millions of people worldwide [72]. The rapid spread of SARS-CoV-2 with many fatal cases is currently considered as global concern. Extensive progress has been making in understanding the virology of SARS-CoV-2 as well as the benefit and drawback of various detection techniques for COVID-19, and the reasons behind the widespread human-to-human transmission of SARS-CoV-2. In addition, there are many other factors contributing to the infectious and pathogenic potential of SARS-CoV-2 that need to be investigated. It is worth noticing that molecular diagnostic tools are crucial for clinical diagnosis, public health monitoring, and mitigation strategies to prevent the spread of COVID-19. Specifically, rapid, simple, affordable, and reliable sensor platforms are currently in demand, especially as the disease spreads across low- and middle-income countries. Even though many emerging platforms have been successfully developed for the accurate diagnosis of COVID-19, including LFAs, plasmonic biosensor, electrochemical sensors, or lab-in-a-chip with the support of nanomaterials, these are not efficient alternatives to RT-PCR technique which is still considered as gold standard diagnostic for confirming the SARS-CoV-2 infection. Table 3 summarizes the advantages and disadvantages of different sensing platforms for detection of SARS-CoV-2, suggesting the possibility of each platform for COVID-19 diagnosis, particularly, LFAs as screening test, NAA-based techniques as confirmatory test, and optical or electrochemical biosensors as tracking test of infection stages and treatment response. Hence, the combination of the different diagnostic techniques could improve the accuracy of COVID-19 detection, significantly improving the effectiveness of diagnosis and treatment. Further advances in analytical technologies through a multidisciplinary approach will strongly associate with the development of therapeutic and vaccine strategies to fight COVID-19 pandemic. Before vaccines are widely approved for clinical use, there was no better way to prevent SARS-CoV-2 infection than through diagnostic tools to monitor public health, and personal preventive behaviors, such as social distancing and masks. For future studies, it is important to continue monitoring the SARS-CoV-2 genome in new cases worldwide to promptly identify any mutations that could lead to changes in the phenotype of the virus. The accurate biosensors have been developed and utilized in clinical diagnosis of COVID-19 after clinical trials, effectively contributing to the better intervention of SARS-CoV-2 pandemic. Thus, the lessons discovered from the COVID-19 molecular diagnostics are valuable for providing a better response to other future diseases. Finally, COVID-19 pandemic is a challenge for all humankind and the management of this pandemic is a permanent undertaking, requiring the efforts of each individual and the international cooperation of scientists, governments, and the public. The satisfactory utilization of advanced diagnostic tools for COVID-19 exhibits the great significance to overcome this pandemic, acquiring the thorough preparation and valuable experiences to win over any future pandemic.

## Figures and Tables

**Table 1:** Advantages and disadvantages of different biomarkers of SARS-CoV-2 as target analytes for COVID-19 diagnosis according to WHO guidelines and various literatures [28,29,30,31,32,33].

No.	Target Analyte	Biosensor Platforms	Advantages	Disadvantages
1	Antigen	Optical sensing, ELISA, lateral flow assay, aptasensing, Lab-in-a Tube sensing system, Lab-on-a Chip sensing system, and Electrochemical sensing.	- Diagnostic tests are usually completed within 30 min. - Detect current infection with high sensitivity and specificity. - Promote to determine which antigen is being developed or commercialized, demonstrating acceptable production in typical field studies.	- Less sensitivity due to no target amplification process. - False positive results if the antibodies also acknowledge antigens from viruses other than SARS-CoV-2. - Depend on the sensitivity and specificity of antigens. - Confirmatory tests should take place
2	Antibody	Optical sensing, ELISA, lateral flow assay, aptasensing, Lab-in-a Tube sensing system, Lab-on-a Chip sensing system, and Electrochemical sensing.	- Maintain an investigation of an in-progress outbreak and supports backdated assessment of the attack rate or size of an outbreak. - Robust and faster in critically ill patients than in patients with milder illness or asymptomatic infection. - No need for immune genetics purification before testing.	- Costly and time-consuming. - Possible only in the recovery phase. - Not indicated for acute diagnosis and clinical administration, and their epidemiological role is under investigation. - Not ensure that these are is neutralizing or protective antibodies. - The lifetime of the antibodies produced in response to SARS-CoV-2 remains to be clarified.
3	RNA	Nucleic acid amplification techniques	- Standard diagnostic test to confirm SARS-CoV-2 infection. - High binding affinity, simple synthesis method, and easy maintenance. - Potential performance benefits, rapid data sharing, as well as urgent regulatory review of possible, well-functioning trials are recommended to increase accessibility to SARS-CoV-2 testing. - Target molecules identified by shape and sequence can be detected more simply.	- False negative results since SARS-CoV-2 continues to have genetic changes over time, misconnected between primers and probes. - RNA should be re-examined by experienced personnel and re-extracted from the original samples. - Swab specimens taken at the late stages of the disease or from the body cavity may not contain virus. - Specimen is not always properly handled and/or transported. - Different viral load in different specimens - Difficulty in genomic diversity and mutations of virus.

**Table 2:** Reported studies of NAA-based techniques and various biosensors for the confirmation of SARS-CoV-2 infection.

No.	Type of Sensing	Detection Platform	Recognition Element	Detection Range/ Qualitative	Limit of Detection (LOD)	Detection Time	Real Sample or Specimens	Analytical/Clinical Sensitivity %	Analytical/Clinical Specificity %	Device/ Commercial Product	Ref.
1	NAA	RT-PCR	RNA	E gene assay: 2.8–9.8 copies/reaction RdRp assay: 2.7–11.2 copies/reaction	E gene assay: 3.9 copies/reaction RdRp assay: 3.6 copies/reaction	25 min	Sputum, nose, and throat swabs	-	-	E gene assay RdRp assay	[35]
2	NAA	PCR	RNA	32.5–1042 copies/mL	100 copies/mL, 242 copies/mL 250 copies/mL 125 genome equivalents/mL	8 h 90 min 45 min 5–15 min	Nasopharyngeal and nasal swab	94 88 100 69	100 100 97 100	Abbott RealTime m2000 SARS-CoV-2 Assay DiaSorin Simplexa COVID-19 Direct Cepheid Xpert Xpress SARS-CoV-2 Abbott ID NOW COVID-19.	[36]
3	NAA	RT-PCR	RNA	Positive and negative	-	-	Oropharyngeal swabs	100 100	100 95.5	MagNA Pure QIAcube	[37]
4	NAA	1-Step Quantitative RT-PCR	RNA	2 × 10^−4^–2000 TCID_50_/reaction	<10 copies/reaction	90 min	Human clinical specimens	-	-	-	[38]
5	NAA	RT-PCR	RNA	Positive and negative	-	-	Human clinical specimens	-	-	-	[39]
5	NAA	RT-LAMP	RNA	Positive and negative	42 copies/reaction	60/90 min	Nasopharyngeal swabs sputum/deep throat saliva throat swab	96.88/98.96 94.03/97.02 93.33/98.33	100 100 100	-	[40]
7	NAA colorimetry	Colorimetric RT-LAMP Swab–to–RT-LAMP without RNA isolation	RNA RNA	Positive and negative	100 RNA molecules/reaction	>30–35 min	Nasopharyngeal swabs	97.5 99.5	99.7 86	-	[41]
8	NAA-Optical	RT-LAMP-LFAs	RNA	1.2 × 10^1^–1.2 × 10^4^ copies per reaction	12 copies/reaction	1 h	Oropharynx swab samples	100	100	-	[42]
9	NAA-Optical	CRISPR-Cas12-based LFAs	RNA	0–25,000 copies/μL	10 copies/μL	40 min	Nasopharyngeal and oropharyngeal swab	-	-	-	[43]
10	Optical	Colorimetric LFAs/ELISA	Antibodies	Positive and negative	-	10/120 min	Serum, plasma	84 65 84 73	99 78 91 96	LFAs Biosynex LFAs Servibio ELISA Euroimmun ELISA EDI	[44]
11	Optical	Colorimetric LFAs	SARS-CoV-2 nucleocapsid protein	- Genemedi N protein: 0.53–0.77 ng/mL. - Genscript N protein: 0.00–7.44 ng/mL	- Genemedi N protein: 0.65 ng/mL - Genscript N protein: 3.03 ng/mL	20 min	-	-	-	Half-Strip LFA	[45]
12	Optical	Colorimetric LFAs	IgM antibody IgG antibody	Positive and negative	-	>15 min	Plasma	50.8 87.3	80 100	Clungene^®^ SARS-CoV-2	[46]
13	Optical	Colorimetric LFAs	IgG antibody	Positive and negative	-	15 min	Serum, plasma, or whole blood	95 91 95 92	98 100 98 100	BTNX kit 1 BTNX kit 2 ACON Laboratories SD BIOSENSOR	[47]
14	Optical	Colorimetric LFAs	SARS-CoV- 2 antibodies	Positive and negative	-	-	Serum specimens	84.4	98.6	LFIAs kít	[48]
15	Optical	Colorimetric LFAs	SARS-CoV-2 nucleocapsid antigen	Positive and negative	-	15–30 min.	Nasopharyngeal and throat swab	98.33	98.73	Standard™ Q COVID-19 Ag kit	[49]
16	Optical	Colorimetric LFAs	IgM/IgG antibody	Positive and negative	-	15 min	Nasopharyngeal swab	100/100 86.36/100 86.36/100 100/100	-	Biotime Biotechnology Co Autobio Diagnostics Co ISIA BIO-Technology Co Biolidics tests	[50]
17	Optical	Electrochemiluminescence immunoassay (ECLIA)	IgG antibody	Positive and negative	-	18–35 min	Serum	92.5 87.5	98.8 97.5	Elecsys^®^ Anti–SARS-CoV-2 LIAISON^®^ SARS-CoV-2 S1/S2 IgG	[51]
18	Optical	Colorimetric/chemiluminescent LFAs	IgA antibody	Positive and negative	-	15 min	Serum, saliva	-	-	-	[52]
19	Optical	Colorimetric LFAs	SARS-CoV-2 antigen	Positive and negative	1.7 × 10^5^ copies/mL	15 min	Nasopharyngeal swab	30.2	100	Coris COVID-19 Ag Respi-Strip test	[53]
20	Optical	ELISA	Neutralizing antibody	Positive and negative	-	-	Blood	-	-	-	[54]
21	Optical	ELISA Pseudovirus neutralization assay Recombinant immunofluorescence assay	IgG antibody IgA antibody	Positive and negative	-	-	Serum	94 90.6	97 85.3	Euroimmun SARS-CoV-2 serological assay	[55]
22	Optical	Plasmonic photothermal biosensor	RNA	0.01 pM to 50 μM	0.22 pM	-	-	-	-	-	[56]
23	NAA-optical	DNA nanoscaffold-fluorescent sensor	RNA	0–100 nM	0.96 pM	10 min	-	-	-	-	[57]
24	NAA-Optical	CRISPR-based Fluorescent assay	RNA	1–10 copies	two copies per sample	50 min	Nasopharyngeal swab	100	71.4	-	[58]
25	Optical	Nanoplasmonic sensor	SARS-CoV-2 virus	10^2^–10^7^ vp/mL	370 vp/mL	15 min	-	-	-	-	[59]
26	Optical	Plasmon-enhanced biosensor	IgM/IgG/IgA	Positive and negative	-	30 min	Serum, direct blood	86.7	100	-	[60]
27	Electrical	Field-Effect Transistor (FET)	SARS-CoV- 2 spike protein SARS-CoV- 2 virus	100 fg/mL–100 pg/mL −10^1^–10^5^ copies/mL	100 fg/mL 2.42 × 10^2^ copies/mL	>1 min	Nasopharyngeal swab	-	-	-	[61]
28	Lab-in-a Tube Optical	Column agglutination test (CAT) technology	Antibodies	Positive and negative	-	10–30 min	Serum specimens	-	-	-	[62]
29	Lab-on-a Chip Optical	Microfluidic fluorescent sensor	IgG/IgM/Antigen	Positive and negative	-	15 min	Serum	-	-	-	[63]
30	Electrochemical	Amperometry	S-RBD protein	0–1400 nM	-	30 s	Nasal secretions and saliva	-	-	-	[64]
31	Electrochemical	Impedance	Antibodies to SARS-CoV-2 S1 protein Receptor-binding-domain (RBD)	1 fM–20 nM 1 fM–20 nM	2.8 ×10^−15^ M 16.9 ×10^−15^ M	10 s	Serum	-	-	-	[65]
32	Electrochemical	Impedance	CR3022 Antibody	0.1–10 µg/mL	-	5 min	Serum	-	-	-	[66]
33	Electrochemical	Amperometry	RNA	585.4–5.854 × 10^7^ copies/μL	6.9 copies/μL	<5 min	Nasopharyngeal swab, saliva	-	-	-	[67]
34	Electrochemical	Differential pulse voltammetry	RNA	10^−17^–10^−12^ M	3 aM	3 h	Sputum, urine, serum, and saliva	-	-	-	[68]

**Table 3:** The advantages and disadvantages of different sensing platforms for SARS-CoV-2 detection.

No.	Platforms/Per-Test Cost	Principle	Advantages	Disadvantages
1	*Nucleic acid amplification* RT-PCR (~50–150 dollars)	Under different temperatures, utilization of a specific set of primers, nucleotides, reverse transcriptase enzyme and DNA polymerase enzyme for reverse transription of RNA into complementary DNA and amplification of cDNA to detect specific target RNA sequence.	- Fairly quick and fewer false-negative results - Higher sensitivity and reliability - Able to follow social distancing when clinical samples are taken from the suspected infected patient’s car or at home. - RT-PCR products are widely available for the detection of clinical samples by medical staff in hospitals or scientists and technicians in laboratories.	- Incapable of completing the detection process in a short time (3–4 h) - Possible to miss corona positive patient who has virus clearance and recovered from disease due to the ability of detection based on capturing and detecting virus. - Costly lab equipment and experimental materials. - Complex detection process but not provide more information about other diseases or symptoms.
	RT-LAMP (~50–150 dollars)	Under isothermal conditions, the utilization of at least two specific sets of primers, nucleotides, reverse transcriptase enzyme and DNA polymerase enzyme for RNA reverse transription and cDNA amplification to detect specific target RNA sequence	- LAMP is more quickly technique that can get results within 1–3 h. - Has a single temperature (60–65 °C) with no specific skills required. - Purification steps are not necessarily based on the stable reaction and inhibitors are tolerated, and results can be recorded with naked eye. - This smaller, simpler, portable method can be performed within hospital laboratories.	- Newer technique that is still being evaluated in clinical. - Too sensitive and susceptible to false positive because of cross-contamination. - Possible to miss corona positive patient who has virus clearance and recovered from disease due to the ability of detection based on capturing and detecting virus. - Not provide more information about other diseases or symptoms.
2	*Optical sensing* Lateral flow assays (~2–10 dollars)	Liquid samples, including target analyte, move without external force through different test trips where molecules that can react to target analyte are captured, resulting in optical signal.	- Remarkably fast for a POC test with final results obtained at approximately less than 30 min. - No need for experts to perform clinical tests, no specialist laboratories or instruments required. - Non-invasive test for the presence of SARS-CoV-2.	- Cannot quantitate the clinical samples. - Intensive experiment to produce antibody - Insufficient evidence for effectiveness and accuracy in SARS-CoV-2 diagnosis is still being evaluated. - Further test should be checked to confirm
	Enzyme-linked immunosorbent assay (~30–70 dollars)	Different antigen-antibody combinations are used, which always include an enzyme-labeled antibody or antigen, and the enzyme activity is measured by optical techniques that collerates with target concentrations.	- Highly sensitive, straightforward, and cheap laboratory technique - High throughput can analyze multiple samples from different patients within 2–4 h. - High-level technicians are not required. - Possibility of quantitating samples. - Well established in hospital	- Not yet well-acknowledged as a standard for SARS-CoV-2 detection. - Intensive experiment to produce antibody. - High probalibity of false positive/negative results - Temporary read-out results in a short timeframe due to the enzyme/substrate reactions.
3	*Electrochemical sensing* (not yet commercialized products)	Due to bio/chemical reaction, the change of bio/chemical signal can translate into electrical signal that collerates with the concentration of target.	- Only a small amount of material is needed. - Simplicity, high sensitivity, consistency, selectivity, and reproducibility. - Provide a faster, real-time detection of target. - Possibility of continuous analysis. - Excellent repeatability with high correctness.	- Identification as prototypes and just evaluation under laboratory conditions so far. - Difficulty in supplying the commercial products. - Narrow or limited temperature range. - Short or limited shelf life. - Difficulty in optimizing the stability, storage, logistics of sensors.

## Data Availability

Not applicable.
